# Interbirth interval practices among reproductive age women in rural and Urban kebeles in Farta Woreda: Case-control study

**DOI:** 10.1371/journal.pone.0256193

**Published:** 2022-01-27

**Authors:** Gedefaye Nibret Mihretie, Simegnew Asmer Getie, Shumye Shiferaw, Alemu Degu Ayele, Tewachew Muche Liyeh, Bekalu Getnet Kassa, Worku Necho Asferie

**Affiliations:** 1 Department of Midwifery, College of Health Sciences, Debre Tabor University, Debre Tabor Town, Ethiopia; 2 Department of Midwifery, College of Health Sciences, Bahir Dar University, Bahir Dar City, Ethiopia; 3 Department of Neonatal Nursing and Child Health, College of Health Sciences, Debre Tabor University, Debre Tabor Town, Ethiopia; Federal University of Sergipe, BRAZIL

## Abstract

**Background:**

Closely spaced births have been reported all over the world especially in developing countries, and they have been correlated with poor maternal and infant health. Enhancing optimal birth interval is one of the key strategies to promote the health status of mothers and their children. However, factors affecting short birth intervals have not been identified in the study area and region. This study was aimed to assess determinants of short birth interval practice among reproductive women in Farta woreda, Ethiopia, 2019.

**Methods:**

Community based unmatched case-control study design was conducted from February to March 2019. The sample size of 303 (101 case and 202 controls) was included by using multistage sampling and then study participants were selected by simple random sampling technique. The data was collected by structured and pre-tested face-to-face interviewer-administered questionnaires from the selected respondents. The collected data were entered with Epi-Data version 4.2 and analyzed by using SPSS version 23 software. Bivariate and multivariate analyses were used to examine the association. Odds ratios, 95% CI, and P-value <0.05 were used to determine the statistical association.

**Results:**

Women who had no formal education (AOR = 2.15, 95% CI (1.19, 3.88), had not a history of antenatal care follow up (AOR = 2.66, 95% CI (1.55, 4.56)), did not use modern contraceptives before getting the latest pregnancy (AOR = 3.48, 95% CI (1.74, 6.95)) and duration of breastfeeding less than 24 months (AOR = 3.59, 95% CI (2.06, 6.24)) were significantly associated with short birth interval.

**Conclusions and recommendation:**

Maternal education, duration of breastfeeding, contraceptive utilization, and antenatal follow-up were identified as the predictor variables of short birth interval practice. Therefore, providing health information for reproductive-age women about the benefit of contraceptive utilization, breastfeeding practice and antenatal care follow up to minimize problems resulting from the short birth intervals.

## Introduction

A short birth interval is an interval between two consecutive births of less than 33 months (birth-to-birth interval). After a live birth, the world health organization recommended intervals before attempting the next birth is at least 33 months to reduce the risk of adverse maternal, perinatal, and infant outcomes [1]. And other studies showed that birth intervals of 3 to 5 years are safer for women and newborns than birth intervals of two years or fewer [2, 3]. Some studies also believe that birth intervals more than five years are less healthy, implying that such mothers may lose the protective advantage of previous childbearing and therefore experience problems similar to primigravida [4].

Closely spaced births have been reported all over the world, and they have been correlated with poor maternal and infant health [5]. In the United States, the study showed that about 33% of pregnancies among women with a previous live birth less than 18 months prior to birth, placing mothers and infants at risk for adverse health outcomes [6]. In addition, women with a short birth interval will not have sufficient time to recover and get ready for the subsequent pregnancy which includes socio-economic, psychological, and physical body preparedness, higher risk for premature rupture of membrane, anemia, failure of a trial of vagina birth after cesarean section (VBAC), post-partum hemorrhage (PPH), preeclampsia, low birth weight, low APGAR (Appearance, Pulse rate, Grimace, Activity and Respiratory rate) score, preterm deliveries were observed [7].

Fertility is an essential component of population dynamics that influences the size and structure of a population [2]. Fertility variables are roughly categorized as direct and indirect. The direct variables are bio-behavioral factors such as sexual activity, contraceptive usage, duration of the postpartum infecund ability, and abortion; whereas, distal determinants, are socio-cultural factors that affect fertility indirectly through affecting the bio-behavioral factors [8, 9].

Birth spacing practices vary greatly throughout the world [10]. Women in developing countries including Sub-Saharan Africa in particular, frequently experience shorter birth intervals than they in developed countries [11].

The study showed that the proportion of short birth interval and median birth interval in Ethiopia was 45.7% [12] and 34.5 months [13], respectively. In Ethiopia there is high proportion of short birth intervals that varies greatly between areas, ranging from 10.6% in Amhara to 45.9% in Somalia [14]. And also, the prevalence of short birth intervals in the Tigray region (Ethiopia) was 23.3% [15], in Debre Birhan Town 40.9% [16].

Short birth intervals have been associated with adverse health outcomes, including infant, child, and maternal mortality [17]. Women with short birth intervals will not have sufficient time to recover in terms of socio-economic, cultural, psychological, and physical body preparedness and get ready for the subsequent pregnancy [18]. Different studies showed that short birth intervals were associated with adverse birth outcomes, including preterm birth, neonatal mortality, low birth weight, small-for-gestational-age, developmental delay, premature rupture of membrane, anemia, postpartum hemorrhage, preeclampsia, and low APGAR score [19–21]. United States Agency for International Development (USAID) study show that closely spaced births have a potentially devastating impact on both the individual and the society and high levels of unplanned fertility, makes it difficult for women to become productive members of society, by limiting their contribution to economic development [22].

Different studies regarding determinant factors of short birth interval revealed that maternal age [23–25], maternal education level [26], husbands’ occupation [27], religion [28], age at first marriage of women [29], duration of breastfeeding [29–32], history of preceding neonatal mortality [28] and contraceptive utilization [27, 32, 33] were found to be significant predictors of short birth interval practice.

Maternal and child complication associated with short birth interval practice has a significant negative impact on socio-cultural and health of the mothers and their children. However, factors affecting short birth interval practice have not been identified and there was scarce studies conducted in the study area at the community level. Therefore, this study aimed to assess the determinant factors of the short birth interval among reproductive-age women who gave birth in the last one year in rural and urban kebeles in Farta, South Gondar Zone, Ethiopia.

## Methods and materials

### Study area and period

This study was conducted from February 30 to March 30, 2019, rural and urban kebeles in Farta woreda, South Gondar, Northwestern Ethiopia. Farta woreda is located 590 kilometers North West of the capital city of Ethiopia, Addis Ababa.

#### Study design

Community based unmatched case-control study design was employed.

#### Source population

Reproductive age women (15–49 years) residing in the study area who experienced at least two consecutive births and the last delivery occurred within the past one year before the data collection.

#### Study population

Reproductive age women residing in selected kebeles in the study area who experienced at least two consecutive deliveries and the last delivery occurred within the past one year before the data collection.

#### Cases

Reproductive-age women who have short birth intervals between the latest two consecutive births.

#### Controls

Reproductive-age women who have optimal births interval between the latest two consecutive births.

### Inclusion and exclusion criteria

#### Inclusion criteria

*Controls*. All women who gave alive birth within the last one year and have at least two consecutive births with a birth intervals of 33 and 59 months between the latest two successive live births.

*Cases*. All women who gave birth within the last one year and have at least two consecutive alive births with a birth interval of fewer than 33 months between the latest two successive live births.

*Exclusion criteria for both cases and controls*. Mothers who had a history of abortion between the last two consecutive births and participants who severed ill.

### Sample size determination

The sample size was determined by using the double population proportion formula for an unmatched case-control study using Open EPI-info version 7 software by taking of contraceptive utilization from the previous study was chosen as an independent variable, since it gives a maximum sample size. The proportion of mothers who did not utilize contraceptives among controls was 33.3% and a minimum detectable OR was considered to be 3.01 [34]. Accordingly, a 5% level of precision, a power of 80%, and a **1:2** allocation ratio of cases to controls were assumed. An additional nonresponse rate of 10% and design effect of 2 was considered. Based on the assumptions, the final sample size was determined to be 303 (101 cases and 202 controls).

### Sampling technique procedure

**Fig 1:**
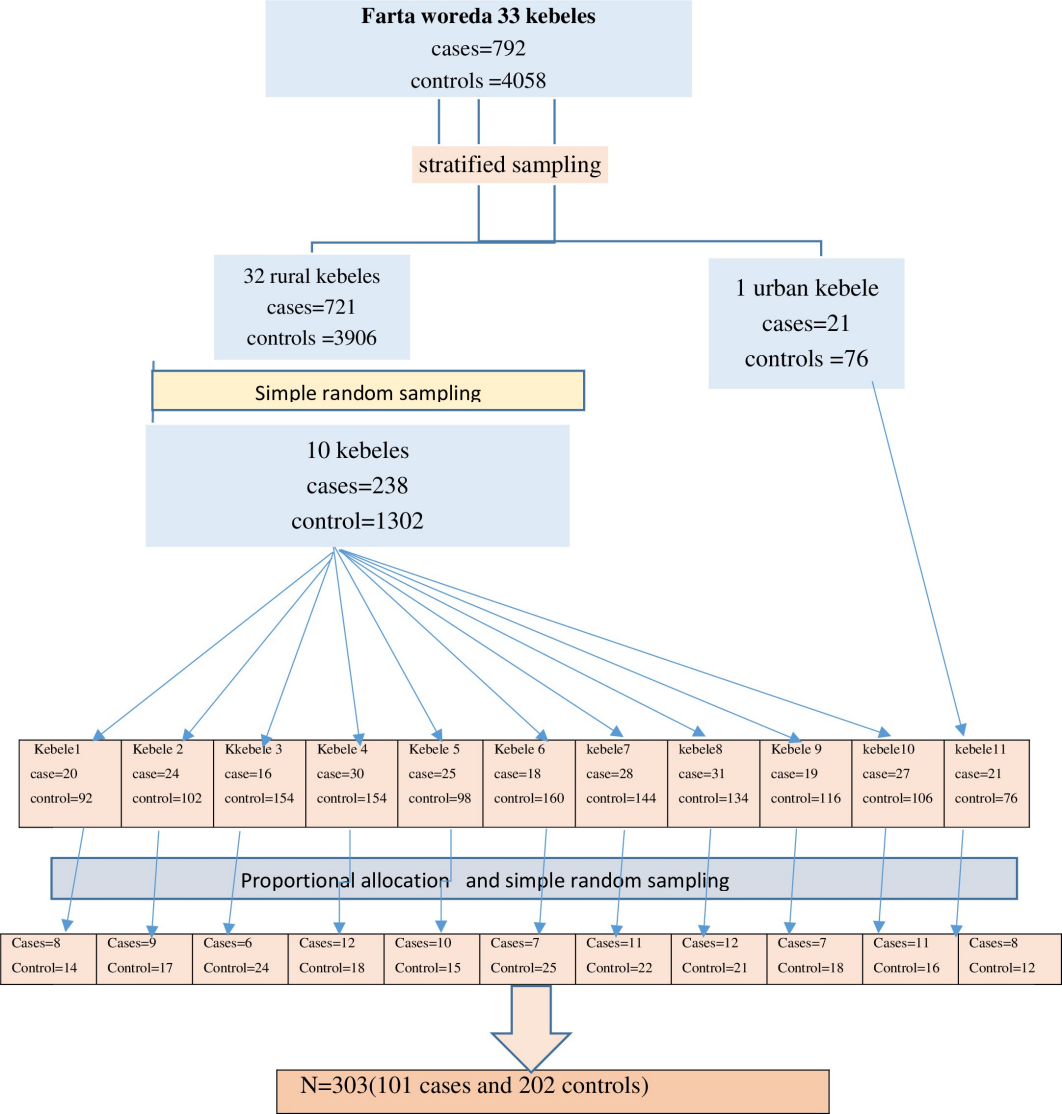
Sampling procedure of short birth interval practice among reproductive age women in Farta woreda, south Gondar zone, North West Ethiopia, 2019.

A multistage sampling technique was used to select study participants. All kebeles (the smallest unit of the district) were first stratified into 1 urban and 32 rural kebeles. Then 1 urban kebele and 10 rural kebeles were selected by simple random sampling methods to get the representative sample size. Then, the sampling frame was prepared from the health extension workers’ registration book (family folders) having household identification numbers. Using respective household identification numbers, frames of households containing study subjects defined as cases and controls were prepared to form each selected kebeles. Then, the proportional allocation technique was employed to determine the study participants from each kebele both for cases and controls. Finally, reproductive-age women who had at least two consecutive births and whose latest birth within the past one year before the data collection. The study participants were selected using a simple random sampling technique from the existing sampling frame by lottery method. The participant who was not present at the time of data collection; three revisits were made to interview the woman. In the case of more than one eligible participant in the household, the lottery method was used to select only one participant (Fig 1).

#### Dependent variables

Short birth interval practice.

#### Independent variables

Socio-demographic variables (age, residency, occupational status of the mother, marital status of the mother, age at first marriage, ethnicity, educational status of the mother, religion of the mother, husband’s educational status, husband’s occupational status). Obstetrics and reproductive health-related variables (age at first pregnancy, gravidity, parity, number of living children, sex of the preceding child, delivery place of the preceding child, survival status of the preceding child, pregnancy plan, antenatal care follows up during previous pregnancy, decision-making power about family planning, Breastfeeding practice, type of contraceptive and contraceptive utilization).

### Operational definitions

#### Short birth interval

When the duration between two consecutive births is less than 33 months [1].

#### Optimum birth interval

When the duration between two successive births is 33 to 59 months [1].

#### Long birth interval

When the duration between two successive births is greater than 59 months [1].

**Birth to birth interval** = birth-to-pregnancy interval(24 months) plus nine months of pregnancy (24+9 = 33 months) [1].

### Data collection tools and procedures

The data were collected by using face-to-face interview-administered, pretested, and structured questionnaires. After reviewing previous literature, the questionnaire was first developed in English and translated to the local language (Amharic) then back to English by language experts to keep its consistency. Five health extension workers who were familiar with the local language and customs were recruited as data collectors and two-degree midwives were assigned as supervisors. The training was given for data collectors and supervisors for two days on data collection procedures, interview techniques, and confidentiality of the information obtained from the respondents. Overall supervision made by the investigators.

### Data quality assurance

Data quality was ensured during data collection, entry, and analysis. Before conducting the main study, a pretest was carried out on 5% of the sample size outside of the selected kebeles and necessary modifications had been made. The principal investigator and supervisor conducted day-to-day on-site supervision during the whole period of data collection. At the end of each day, the questionnaires were reviewed, checked for completeness and accuracy by the supervisor and investigator. Then, corrective discussions were undertaken by the research team members.

### Data analysis

After coding, the data were entered into Epi-Data version 4.2 and then exported to SPSS version 23 software for analysis. Bivariate analysis was executed to examine the crude association of predictors with short birth intervals. Finally, variables having p-values ≤0.2 on bivariate analysis were selected as candidates for multivariable analysis. Odds Ratio and 95% CI and P-values less than 0.05 were used to determine the statistical significance of the tests. Finally, the results were presented in texts and tables.

### Ethical consideration

After approval, ethical clearance was obtained from the Institutional Review Board (IRB) of Medicine and Health Sciences, Bahir Dar University. A permission letter was obtained from the Farta woreda health office before starting data collection. At the beginning of the data collection, written informed consent was obtained from each respondent after a thorough explanation of the purpose and the procedures of the study. Respondents were also informed that all the data obtained from them were kept confidential and anonymous. To ensure confidentiality, the names of respondents were replaced by code numbers.

## Results

### Socio-economic characteristics of participants

This study was carried out with a total of 303 child-bearing age women who had at least two consecutive births with a response rate of (99%). The mean age of the respondents was 33.5 (SD±5.5) and 33.9 (SD ± 5.5) years among cases and controls, respectively. Among the respondents, 99% were Amhara in ethnicity. About 97% of the cases and 95.0% of the controls were married. Regarding educational status, 73 (73.0%) of the cases and 58% of controls did not attend formal education. Concerning religion, 98.0% cases and 98.5% of controls were Orthodox Christian followers. Eighty-four percent of the cases’ husbands and 85.0% of the controls husbands’ occupation were farming (Table 1).

**Table 1:** Socio-demographic characteristics of reproductive age mothers (N = 300) in Farta Woreda, South Gondar Zone, North West, Ethiopia, 2019.

Variables	Case(n = 100)		Control(n = 200)	
	Frequency	Percent (%)	Frequency	Percent (%)
Maternal age				
20–25	8	8.0	13	6.5
26–30	25	25.0	53	26.5
31–35	29	29.0	47	23.5
36–40	26	26.0	65	32.5
41–45	12	12.0	22	11.0
Marital status				
Married	97	97.0	190	95.0
Others*	3	3.0	10	5.0
Age at first marriage				
12–18	46	46.0	97	48.5
19–24	47	47.0	88	44.0
25–29	7	7.0	15	7.5
Residency				
Urban	8	8.0	21	10.5
Rural	92	92.0	179	89.5
Religion				
Orthodox	98	98.0	197	98.5
Others**	2	2.0	3	1.5
The educational level of mothers				
No formal education	73	73.0	116	58.0
Has formal education	27	27.0	84	42.0
Mothers’ occupation				
Housewife	70	70.0	115	57.5
Farming	21	21.0	66	33.0
merchant	9	9.0	19	9.5
Husbands’ educational level				
No formal education	52	52.0	82	41.0
Has formal education	48	48.0	118	59.0
Husband’s occupation				
Farming	84	84.0	170	85.0
Daily laborer	8	8.0	13	6.5
Merchant	8	8.0	17	8.5

Others* = Single/widowed/divorced, others** = Muslim, protestant

### Obstetrics and reproductive health history of respondents

The mean duration of the birth interval was 25.36 with SD ± 4.37 and 44.86 ± 7.83 months among cases and controls, respectively. Fifty percent of the cases and ninety-four (47.0%) controls were reported to have three up to four alive children. Eight percent of the cases and nine percent of the controls had a history of stillbirth before the preceding birth.

Thirty-two percent of the case and 43.5% of the controls gave birth in health institutions for their preceding child. A majority (73.0%) of the cases breastfed for less than 24 months, while one hundred twenty (60.0%) of the controls breastfed for 24 months or above. Seventy percent of the cases and one hundred seventy-nine (89.5%) of the controls utilized modern contraceptive methods after the delivery of the preceding child but before they got pregnant for the last child. Fifty-seven (57.0%) of cases and seventy-one (35.5%) of controls did not have antenatal care visits during their latest pregnancy (Table 2).

**Table 2:** Obstetric, breastfeeding, and contraception history of reproductive age mothers (N = 300) in Farta Woreda, South Gondar Zone, North West, Ethiopia, 2019.

Variables	Case(n = 100)		Control(n = 200)	
	Frequency	Percentage (%)	Frequency	Percentage (%)
Age at first pregnancy				
12–18	16	16.0	46	23.0
19–24	72	72.0	139	69.5
> = 25	12	12.0	15	7.5
Number of living children				
0–2	28	28.0	43	21.5
3–4	50	50.0	94	47.0
> = 5	22	22.0	63	31.5
Abortion before the preceding child				
Yes	10	10.0	39	19.5
No	90	90.0	161	80.5
Still, birth before preceding child				
Yes	8	8.0	18	9.0
No	92	92.0	182	91.0
Sex of the preceding child				
Female	60	60.0	90	45.0
Male	40	40.0	110	55.0
Status of the preceding Child				
Alive	94	94.0	185	92.5
Dead	6	6.0	15	7.5
Last child pregnancy plan				
No	20	20.0	21	10.5
Yes	80	80.0	179	89.5
ANC in preceding pregnancy				
No, follow up	57	57.0	71	35.5
One follow up	19	19.0	57	28.5
Two follow up	11	11.0	40	20.0
Three follow up	9	9.0	20	10.0
Four and above	4	4.0	12	6.0
Place of the previous delivery				
Health institution	32	32.0	87	43.5
Home	68	68.0	113	56.5
Contraceptive use before latest pregnancy				
No	30	30.0	21	10.5
Yes	70	70.0	179	89.5
Type of contraceptive(n = 251)				
Depo-Provera	62	89.9	153	85.5
Implant	5	7.2	21	11.7
Others*	2	2.9	5	2.8
Decision-maker about F/P			1	
Self (Mother)	53	53.0	78	39.0
Both husband and wife	43	43.0	115	57.5
Husband only	4	4.0	7	3.5
Duration of Breastfeeding				
<24 months	73	73.0	80	40.0
> = 24months	27	27.0	120	60.0

Others*, oral pills, emergency pills, male condom.

### Determinant factors affecting short interbirth interval practice

First bi-variable logistic regression was conducted among twenty-one variables only eleven variables with p-values of 0.2 or less were included in the multivariable logistic regression. These variables were: educational level of mothers, husbands’ educational level, age at first pregnancy, number of living children, sex of the preceding child, the status of the preceding child, latest pregnancy plan, ANC in preceding pregnancy, place of previous delivery, contraceptive use between preceding birth and latest pregnancy and duration of breastfeeding.

As can be depicted from multivariable backward stepwise logistic regression mothers who did not have formal education, did not have modern contraceptive methods before getting pregnant for the latest child, breastfed for the preceding child less than 24 months, and mothers who have no antenatal care follow up in preceding pregnancy were found to be significantly associated with short birth interval

Mothers with no formal education were about 2 times (AOR = 2.15, 95% CI (1.19, 3.88)), more likely to have short birth intervals as compared to those who attended formal education. The odds of having a short birth interval were higher among mothers who did not use modern contraceptive methods before getting pregnant for the last child (AOR = 3.48, 95% CI (1.74, 6.95)) than those who had a history of using modern contraceptives. Mothers who breastfed for the preceding child for less than 24 months were 3 times more likely to have short interbirth intervals than their counterparts of mothers who breastfed for 24 months or more (AOR = 3.59, 95% CI (2.06, 6.24)). The odds of having a short birth interval were higher for mothers who hadn’t a history of antenatal care follow-up in preceding pregnancy than those who didn’t have (AOR = 2.66, 95% CI (1.55, 4.56)) (Table 3).

**Table 3:** Bivariate and multivariable analysis of determinant factors among reproductive-age women in Farta Woreda, South Gondar Zone, North West, Ethiopia, 2019.

Variables	Cases(n = 100) Number (%)	Controls(n = 200) Number (%)	COR (95% CI)	AOR (95% CI)	P-values
The educational level of mothers					
No formal education	73(73.0)	116(58.0)	1.95(1.15,3.28)	2.15(1.19,3.88)	.011
Had formal education	27(27.0)	84(42.0)	1		
Husbands’ educational level					
No formal education	52(52.0)	82(41.0)	1.55(.96,2.52)	.66(.37,1.16)	.154
Has formal education	48(48.0)	118(59.0)	1		
Age at fist pregnancy					
12–18 years	16(16.0)	46(23.0)	1		
18 -24years	72(72.0)	139(69.5)	1.48(.78, 2.81)	1.23(.60, 2.54)	.565
> = 25	12(12.0)	15(7.5)	2.30(.89, 5.93)	2.20(.73, 6.56)	.157
Number of living children					
0–2	28(28.0)	43(21.5)	1.86(.94, 3.68)	.829(.42, 1.63)	.587
3–4	50(50.0)	94(47.0)	1.52(.84, 2.76)	.58(.26,1.280)	.180
> = 5	22(22.0)	63(31.5)	1		
Sex of the preceding child					
Female	60(60.0)	90(45.0)	1.83(1.12,2.98)	1.29(.72, 2.31)	.385
Male	40(40.0)	110(55.0)	1		
Status of the preceding Child					
Alive	94(94.0)	185(92.5)	1		
Dead	6(6.0)	15(7.5)	.78(.29,2.09)	.98(.33,3.13)	.970
Last pregnancy plan					
No	20(20.0)	21(10.5)	2.13(1.09,4.15)	1.70(.81, 3.57)	.160
Yes	80(80.0)	179(89.5)	1		
ANC in preceding pregnancy					
No	59(59.0)	71(35.5)	2.61(1.59, 4.27)	2.66(1.55,4.56)	.001
Yes	41(41.0)	129(64.5)	1		
Place of the previous delivery					
Health institution	32(32.0)	87(43.5)	1		
Home	68(68.0)	113(56.5)	1.63(.98, 2.71)	1.43(.77, 2.63)	.250
Contraceptive use					
No	30(30.0)	21(10.5)	3.65(1.96,6.80)	3.48(1.74,6.95)	.001
Yes	70(70.0)	179(89.5)	1		
Duration of Breast feeding					
<24 months	73(73.0)	80(40.0)	4.05(2.38,6.79)	3.59(2.06,6.24)	.001
≥ 24 months	27(27.0)	120(60.0)	1		

## Discussion

Short birth interval is critical for many Sub-Saharan African countries including Ethiopia, where perinatal mortality and fertility remain high. However, birth spacing was affected by different factors. Thus, this community-based case-control study identified factors influencing short birth interval practice among childbearing age mothers.

In this study, the odds of having a short interbirth interval were higher among mothers who did not have formal education as compared to participants who attended formal education. This finding was similar to studies conducted in Arba Minch, Pakistan, Mekele, Ethiopian DHS 2011, and Iran [34, 35]. This might be due to women with formal education are more likely to have better knowledge and decision-making ability for using modern contraceptives and other reproductive health services. Additionally, educated women might have better access to print media exposure regarding the importance of optimal birth interval practice.

Women who did not have a history of modern contraceptive utilization before they got pregnant for their latest child were more likely to experience short birth intervals than women who had a history of contraceptive utilization. This was similar to studies done in Arba Minch, southern Ethiopia, Lemo district, Manipur, and Mekele [26, 27, 32, 36] respectively. This might be explained by women who had a history of using modern contraceptives will increase the risk of unintended pregnancy which leads to closely spacing birth intervals.

Women who breastfed the preceding child for less than 24 months were more likely to have short interbirth intervals than participants who breastfed for 24 months and above. This finding was similar to the studies done from Arba Minch, southern Ethiopia, Manipur, Nigeria, Mekele [29, 31]. This may be since breastfeeding extends the period of the inter-birth interval through negative hormonal feedback. Breastfeeding leads to the secretion of prolactin hormone from the pituitary gland and lowers follicular stimulating hormone and luteinizing hormone levels in the blood. Subsequently, ovulation is delayed and the amenorrhea period prolongs, reducing the risk of fertility [37].

Women who had a history of antenatal care follow-up were more likely to have optimal birth interval practice than women who didn’t attend antenatal care follow-up during the preceding pregnancy. This might be due to women with a history of ANC follow up might get information from health care providers about family planning and the bad outcomes of short birth interval practice.

### Strength

Community-based study design.

### Limitation

Since the study participants include within one year after birth memory bias might be introduced and this study focuses on a Quantitative approach Which could not address the “why” questions in detail.

## Conclusion and recommendation

In this study educational status of the mother, contraceptive utilization, duration of breastfeeding and antenatal care follows up in preceding pregnancy were determinant factors for short birth interval practice. Special attention should be given to the women by giving health information about the benefits of contraceptive utilization, the benefit of breastfeeding for birth spacing, family planning counseling during antenatal care follow-up at the community level as well as encouraging women for education.

## Abbreviations

ANC: Antenatal Care
APGAR: Appearance, Pulse rate, Grimace, Activity and Respiratory rate
AOR: Adjusted odds ratio
COR: crude odds ratio
CI: confidence interval
WHO: world health organization
